# Geological Borehole Video Image Stitching Method Based on Local Homography Matrix Offset Optimization

**DOI:** 10.3390/s23020632

**Published:** 2023-01-05

**Authors:** Zhaopeng Deng, Shengzhi Song, Shuangyang Han, Zeqi Liu, Qiang Wang, Liuyang Jiang

**Affiliations:** College of Information and Control Engineering, Qingdao University of Technology, Qingdao 266525, China

**Keywords:** image stitching, image matching, homography matrix, feature detection, borehole panorama

## Abstract

Due to the influence of the shooting environment and inherent image characteristics, there is a large amount of interference in the process of image stitching a geological borehole video. To accurately match the acquired image sequences in the inner part of a borehole, this paper presents a new method of stitching an unfolded borehole image, which uses the image generated from the video to construct a large-scale panorama. Firstly, the speeded-up robust feathers (SURF) algorithm is used to extract the image feature points and complete the rough matching. Then, the M-estimator sample consensus (MSAC) algorithm is introduced to remove the mismatched point pairs and obtain the homography matrix. Subsequently, we propose a local homography matrix offset optimization (LHOO) algorithm to obtain the optimal offset. Finally, the above process is cycled frame by frame, and the image sequence is continuously stitched to complete the construction of a cylindrical borehole panorama. The experimental results show that compared with those of the SIFT, Harris, ORB and SURF algorithms, the matching accuracy of our algorithm has been greatly improved. The final test is carried out on 225 consecutive video frames, and the panorama has a good visual effect, and the average time of each frame is 100 ms, which basically meets the requirements of the project.

## 1. Introduction

With the vigorous development of mining, tunnel construction, oil mining and other engineering projects, the importance of geological structure analysis is self-evident. Due to the popularization of optical technology [1], camera technology has been applied for geological exploration. Among them, forward-looking borehole camera technology can be directly used to measure the inner wall of the borehole to obtain better information about the rock mass structural plane, and it is not affected by the drilling coring process. It has become one of the important methods in geological exploration [2]. The borehole video captured using the axial view panoramic borehole camera system (APBCS) can be converted into a borehole wall unfolded image sequence, and a complete borehole panorama that intuitively reflects the characteristics of the borehole wall’s structural plane can be constructed by using image stitching method [3]. Using the panorama, the trend, crack width, rock mass interface and other information about the rock mass structural plane can be analyzed from the macro perspective, which provides a research basis for further qualitative analyses of borehole data. It has important theoretical significance and practical application value.

In recent years, image stitching technology has been widely used in the military, agriculture, geological exploration and other fields [4,5], and it has gradually become an important branch of image processing [6,7,8]. Image matching is at the core of image stitching [9,10], and its essence is to calculate the geometric transformation relationship between two images using overlapping areas to obtain the rotation, scale, displacement and other parameter values, and then realize the mosaic of the overlapping areas [11,12]. At present, the mainstream image matching method is the feature-based matching method [13]. Lowe (2004) [14] put forward the scale-invariant feature transform (SIFT) algorithm in 2004, which shows good robustness in image translation, scaling and rotation. However, it requires a lot of operating, resulting in image stitching taking a long time. Subsequently, the speeded-up robust features (SURF) algorithm, which is improved from SIFT, was introduced to enhance the operating efficiency of the algorithm by taking the derivative of the integral image Haar wavelet (Haar) [15].

In the process of image stitching, some scholars have proposed many image matching algorithms based on feature points in combination with the application characteristics of different fields [16]. Chen et al. [17] added a nonrigid matching algorithm based on VFC on the basis of SIFT to make the matching between remote sensing images taken by UAVs more accurate. However, due to the complexity of the SIFT algorithm itself, the introduction of other algorithms for fusion resulted in running times that were too long, which made them inapplicable to some real-time stitching systems, and they could not meet the requirements of rapidity. Yuan et al. [18] proposed a seamless stitching method of UAV aerial images by combining the adaptive as-natural-as-possible (AANPA) method with the proposed energy function. It can effectively eliminate cracks in the stitching of UAV images and make the stitched obtained images more accurate and comprehensive. However, the stitching applicability between images with a small number of feature points and relatively small shape variables remains to be verified. Bai et al. [19] proposed a video mosaic method of coal mine monitoring based on feature points, which introduces the Moravec corner detection algorithm on the basis of the SIFT and RANSAC algorithms to solve the problem of feature re-extraction due to the change in the observation angle, but the algorithm efficiency needs to be further improved.

The borehole unfolded image sequence studied in this paper was taken from inside the geological borehole using the APBCS device. Due to the particularity of the geological environment, the acquired rock mass image has a relatively singlemonotone color, texture, structure and other features, which will produce many mismatched points during feature extraction and matching [20], resulting in inaccurate image transformation which will affect the accuracy of the image stitching. To solve the above problems, this paper proposes a new stitching method for the geological borehole unfolded image sequence. We use the SURF and MSAC algorithms to obtain the homography matrix and present a local homography matrix offset optimization (LHOO) algorithm to obtain the optimal offset. By repeating the above process frame by frame, we can realize the continuous stitching of the unfolded image sequence, effectively improve the matching accuracy, and provide conditions for the quantitative analysis of specific borehole wall information data, which increases the value of forward-looking borehole camera systems used in geological explorations.

## 2. Overview of the System

**Figure 1:**
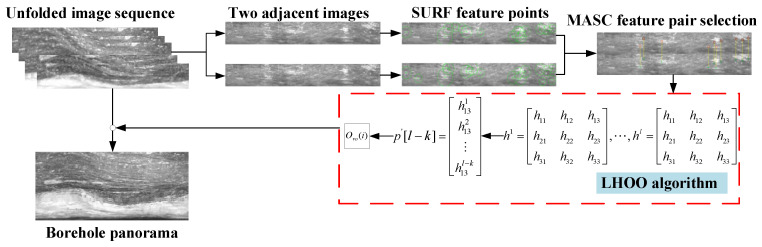
Flow chart of the video image stitching system.

According to the particularity of the geological borehole video captured using the APBCS, this paper proposes a geological borehole video image stitching method based on the local homography matrix offset optimization (LHOO) algorithm. The system architecture of our stitching method based on LHOO is illustrated in Figure 1.

Two previous frames of the borehole unfolded image sequence are taken as the primary stitching task. Firstly, the image feature points are extracted by the SURF algorithm to complete the rough matching task. Then, the MSAC algorithm is introduced to remove the mismatched point pairs and obtain the homography matrix [21]. Subsequently, in the proposed LHOO algorithm, the local parameters of the homography matrix are used to obtain the vertical offset of two consecutive unfolded images, and the optimal offset is obtained by using the statistical optimization algorithm of multiple offsets proposed in this paper. Finally, the above method is used for the continuous stitching of the unfolded image sequence to obtain a complete borehole panorama.

## 3. Methodology

The stitching process of borehole unfolded image sequence mainly includes feature matching and image stitching [22,23]. The essence of feature matching is to obtain eigenvector descriptors through using the feature point detection algorithm, then, we use the extracted parameters for rough matching and fine matching to obtain the transformation matrix between the images. The image stitching involves splicing two images with overlapping parts into a large-scale complete image based on the transformation matrix [24].

### 3.1. Rough Matching of Borehole Unfolded Image

#### 3.1.1. SURF Feature Point Extraction

SURF, which is a popular algorithm that has been used for image registration in recent years, is a fast and robust local feature point description algorithm. It is mainly used in the field of machine vision, such as object detection, target recognition and 3D reconstruction and so on [25], which is and it can be divided into the following five stages.

(1) Generate an integral image

The size of the integral image *P*_∑ (*x*, *y*)_ is consistent with that of the source image *P* (*x*, *y*). We draw the coordinate axis, with the lower left corner of the image being the origin, and sum the pixel values of horizontal and vertical coordinates from the (0, 0) point to the (*x*, *y*) point as the integral value at (*x*, *y*), as shown below:

(1) $$P_{\sum \left(x,y\right)}=\sum_{i\leq 0}^{i\leq x}\sum_{i=0}^{j\leq y}P(x,y)$$

(2) Build a scale space pyramid

The Gaussian kernel is replaced by the box filter with the SURF algorithm, and then, the scale space is formed by convolution with the expanded box filter template and the original image. We can change the size of the box filter template to obtain the corresponding scale image.

(3) Locate the key extreme points

After building the scale space, all of the pixels in the response image are calculated by the determinant of Hessian and compared with 26 pixels in the 3D neighborhood of the current image. If the value of the point is a maximum, then it is retained as a feature point, as shown in Equation (2):

(2) $$\det(H_{approx})=D_{xx}D_{yy}-{\left(0.9D_{xy}\right)}^{2}$$

where, *D_xx_*, *D_yy_* and *D_xy_* are the second derivative values of the corresponding direction.

(4) Direction matching

The feature point is the centre of the circle which is used to draw a circular region with a diameter of 6 *s*; *s* is the scale. In this area, we count the sum of the Haar wavelet features of all of the feature points in the 60-degree sector area and rotate them around the current point in steps of 15 degrees. The maximum area of the module length is the current point direction.

(5) Feature description

A square neighbourhood is constructed around the feature points with a side length of 20 *s*, and the neighborhood direction is the main direction in the fourth step. To form the SURF descriptors, the neighborhood is equally divided into a 4 × 4 sub-region to count the Haar wavelet characteristics at horizontal and vertical directions in all of the sub-regions [26].

(3) F=(∑dx,∑dy,∑|dx|,∑|dy|)

#### 3.1.2. Feature Point Matching Based on the Euclidean Distance

The Euclidean distance is calculated for all of the feature points of the pre-matched image. The smaller the value is, the higher the matching degree of corresponding feature points is. In addition, the SURF algorithm counts the positive and negative relationships of the Hessian matrix trace of the current feature point. If the signs of the trace value of the two feature points are opposite to one another, this feature point pair will be excluded. The basis of the judgement basis of the feature point pairs that is based on the Euclidean distance is as follows [27]:

(4) $$D(i,j)={\left[\sum_{k=1}^{n}{\left(D_{ik}-D_{jk}\right)}^{2}\right]}^{\frac{1}{2}}$$

where *D* (*i*, *j*) is the Euclidean distance of the eigenvector between point *i* in the matching image and point *j* in the template image. *N* represents the dimension of the characteristic vector, *D_ik_* is the *k*-th characteristic component of point *i*, and *D_jk_* is the *k*-th characteristic component of point *j*.

#### 3.1.3. Homography Matrix Extraction Based on MASC Algorithm

The MSAC (M-estimate sample consensus) algorithm is an improved algorithm based on RANSAC (random sample consensus) [28,29]. The specific implementation steps are as follows:

(1) According to the properties of the homography matrix, it is necessary to randomly select 4 pairs of matching points based on the Euclidean distance from the rough matching point pairs and calculate the current homography matrix by using the reverse inference method, as follows:

(5) $$s\left[\begin{array}{c} x^{\prime }\\ y^{\prime }\\ 1 \end{array}\right]=\left[\begin{array}{ccc} h_{11} & h_{12} & h_{13}\\ h_{21} & h_{22} & h_{23}\\ h_{31} & h_{32} & h_{33} \end{array}\right]\left[\begin{array}{c} x\\ y\\ 1 \end{array}\right]$$

where *s* is the scale, (*x*, *y*) is the feature point position of the image to be fused, and (*x*’, *y*’) is the feature point position of the source image.

(2) We can use the homography matrix to calculate the symmetrical transformation error for the rest of the matching point pairs [30,31]. The points, whose values are less than the threshold value, are considered as interior points, as follows:

(6) $$\|\left[\begin{array}{c} x_{i}^{\prime }\\ y_{i}^{\prime }\\ 1 \end{array}\right]-H\left[\begin{array}{c} x_{i}\\ y_{i}\\ 1 \end{array}\right]\|\leq t$$

(3) After counting the number of interior points, if the number of interior points corresponding to the current transmission projection matrix is the largest one, the model is considered to be the optimal model [32].

Compared with the RANSAC algorithm, the MSAC algorithm overcomes the shortcomings of the RANSAC algorithm, which is sensitive to the threshold value, to ensure the stability of the algorithm, and the MSAC algorithm can reflect not only the number of model data, but also the degree of data fitting.

### 3.2. Local Homography Matrix Offset Optimization Algorithm (LHOO)

**Figure 2:**
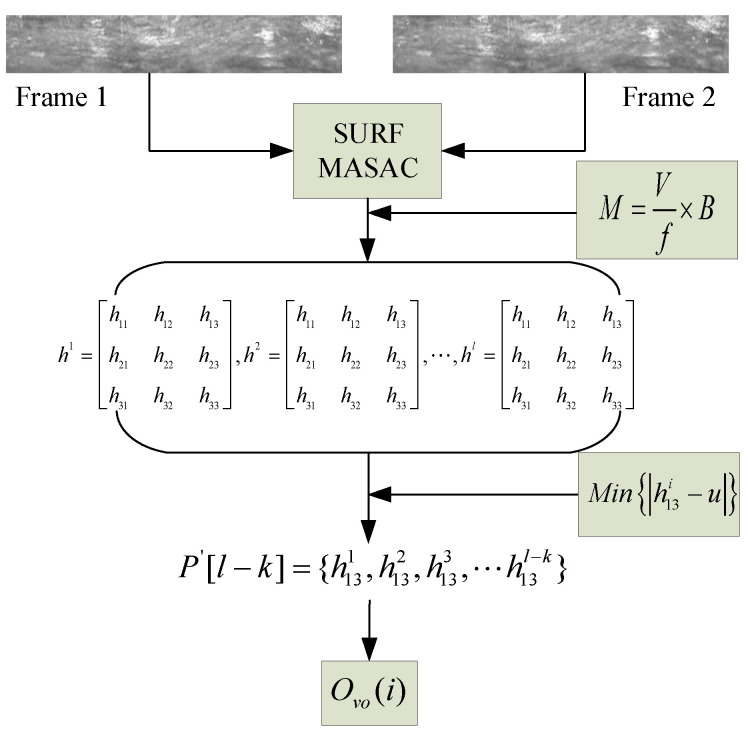
LHOO algorithm flowchart.

As it is the most important parameter in the process of video image mosaic, the accuracy of the offset of the unfolded image sequence directly affects the precision of the generated borehole panorama. In this paper, the features of the geological borehole video image are relatively simple, and the there is a large amount of interference in the accurate matching of the feature points. In addition, due to the particularity over the course of taking the picture, the vertical offset value of image sequence is much higher than the horizontal offset. In view of the above characteristics, this paper proposes a new LHOO algorithm to obtain the optimal vertical offset to ensure the accuracy of the video image stitching process. Its flowchart is shown in Figure 2, and the implementation steps are as follows:

(1) Obtain multiple homography matrices

The current image group is iterated repeatedly based on the SURF and MSAC algorithms to obtain *l* homography matrices.

(7) $$h^{1}=\left[\begin{array}{ccc} h_{11} & h_{12} & h_{13}\\ h_{21} & h_{22} & h_{23}\\ h_{31} & h_{32} & h_{33} \end{array}\right],h^{2}=\left[\begin{array}{ccc} h_{11} & h_{12} & h_{13}\\ h_{21} & h_{22} & h_{23}\\ h_{31} & h_{32} & h_{33} \end{array}\right],\cdots ,h^{l}=\left[\begin{array}{ccc} h_{11} & h_{12} & h_{13}\\ h_{21} & h_{22} & h_{23}\\ h_{31} & h_{32} & h_{33} \end{array}\right]$$

(2) Evaluate the threshold of vertical offset

According to the video frame rate and the camera displacement rate, we roughly calculate the number of inter frame strokes of the shooting platform, and it is multiplied by the offset coefficient to obtain the vertical offset threshold:

(8) $$M=\frac{S}{f\times t}\times B=\frac{v}{f}\times B$$

where *M* is the threshold of vertical offset, *S* is the platform displacement in *t* seconds, *f* is the video capture frame rate, *v* is the camera displacement rate, and the offset coefficient *B* is estimated from the lens focal length, image proportion and camera specifications, etc. Based on the speed of the camera equipment traveling in the borehole and a series of experiments testing, we finally determined the value of *M* as 15.

(3) Build the array that is to be filtered

We extract *l* local vertical offsets *h*_13_, corresponding to multiple homography matrices in the first step to obtain the vertical offset array. If the current offset *h*_13_ is less than *M*, the value will be rounded and stored in the new array *P*’[*l* − *k*]. If *h*_13_ is greater than *M*, the current homography matrix will be removed. The final array *P*’ that is to be filtered is composed of a one-dimensional array, whose size is *l* − *k*.

(9) $$P^{\prime }[l-k]=\{h_{13}^{1},h_{13}^{2},h_{13}^{3}\cdots ,h_{13}^{l-k}\}$$

where *k* is the number of homography matrices which has finally been eliminated.

(4) Extract the optimal vertical offset data set

We calculate the Pearson mode (PM) from *P*’ as the initial cluster center μ and obtain the Euclidean distance from all of the offsets in *P*’ to the *μ* in the proper order ($\left|h_{13}^{i}-u\right|$), and then, we select the offset corresponding to the minimum value as the optimal vertical offset, which is expressed as:

(10) $$O_{vo}(i)=Min\left\{\left|h_{13}^{i}-u\right|\right\}=Min\left\{\left|h_{13}^{i}-\left[\varepsilon -3\left(\varepsilon -M_{d}\right)\right]\right|\right\}$$

where *ε* and *M_d_* are the mean and median values of the array, respectively.

After cycling the above steps frame by frame, the data set of stitching parameters is generated, which is composed of the optimal vertical offsets (*O_vo_*) between the adjacent borehole unfolded images, i.e., {*O_vo_*(1), *O_vo_*(2), …, *O_vo_*(N)}.

To sum up, the pseudo-code of the image matching based on the LHOO is summarized in Algorithm 1.
**Algorithm 1.** Pseudo-code: LHOO algorithm**Input:** Image sequence to be stitched {F(1), F(2), F(3), …, F(N)} **Output:** Data set of stitching parameters {*O_vo_*(1), *O_vo_*(2), …, *O_vo_*(N)}
**Obtain:***l* homography matrices of the current image group F(1) and F(2) based on the SURF and MSAC$h^{1}=\left[\begin{array}{ccc} h_{11} & h_{12} & h_{13}\\ h_{21} & h_{22} & h_{23}\\ h_{31} & h_{32} & h_{33} \end{array}\right],h^{2}=\left[\begin{array}{ccc} h_{11} & h_{12} & h_{13}\\ h_{21} & h_{22} & h_{23}\\ h_{31} & h_{32} & h_{33} \end{array}\right],\cdots ,h^{l}=\left[\begin{array}{ccc} h_{11} & h_{12} & h_{13}\\ h_{21} & h_{22} & h_{23}\\ h_{31} & h_{32} & h_{33} \end{array}\right]$**Set:** Threshold of vertical offset (*M*)**Compute:**$M=\frac{v}{f}\times B$Build the array *P*’[*l* − *k*]**if** $h_{13}^{i}<M$, the value will be rounded and stored in *P*’**Obtain:**$P^{\prime }[l-k]=\{h_{13}^{1},h_{13}^{2},h_{13}^{3}\cdots ,h_{13}^{l-k}\}$**Compute:** Optimal vertical offset**for** *i* = 1 to *l* − *k*  $O_{op}(i)=Min\left\{\left|h_{13}^{i}-u\right|\right\}=Min\left\{\left|h_{13}^{i}-\left[\varepsilon -3\left(\varepsilon -M_{d}\right)\right]\right|\right\}$**end for****Obtain:***O_vo_*(*i*)**for** *i* = 1 to *N*Cycle 1 to 12 steps frame by frame**end for****Obtain:** Data set of stitching parameters {*O_vo_*(1), *O_vo_*(2), …, *O_vo_*(N)}


### 3.3. Generate the Cylindrical Borehole Panorama

**Figure 3:**
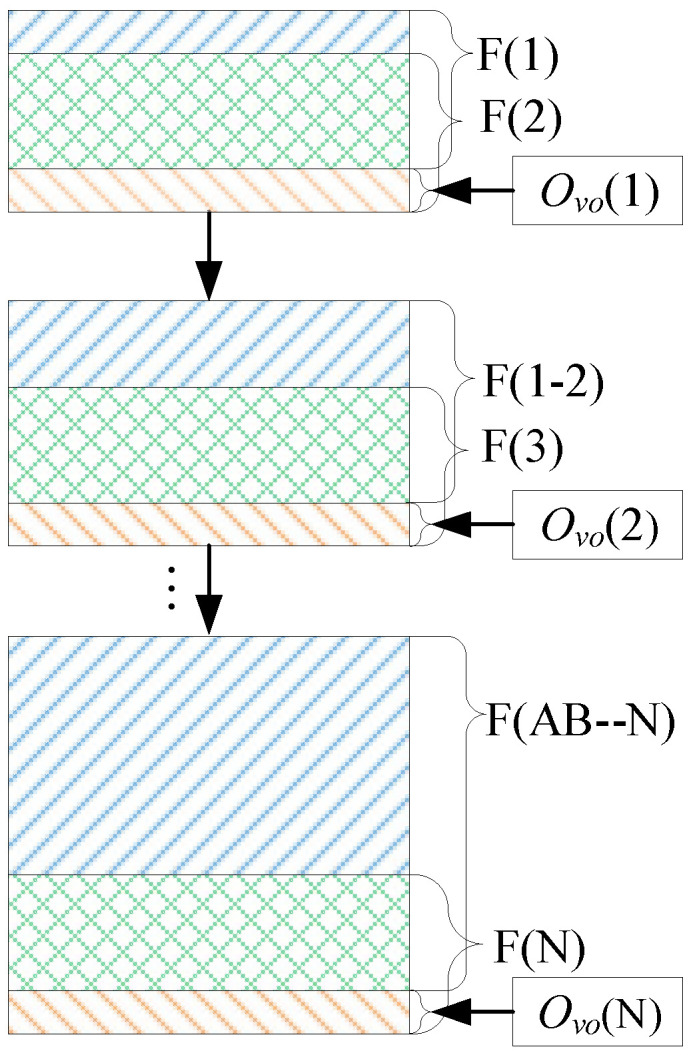
Generation process of cylindrical panorama.

After using the LHOO algorithm proposed in this paper to obtain the data set ({*O_vo_*(1), *O_vo_*(2), *O_vo_*(3), …, *O_vo_*(N)}) which is composed of the optimal vertical offset between the video frame sequences, we use the borehole unfolded image sequence ({F(1), F(2), F(3), …, F(N)}) to construct a cylindrical borehole panorama. According to the optimal vertical offset *O_vo_*(*i*), the two adjacent unfolded images are stitched one by one to generate a complete panoramic image, and the steps are as follows:

Firstly, the first image F(1) and the second image F(2) are spliced into one image F(1-2) by using the optimal vertical offset *O_vo_*(1). Then, the images F(1-2) and F(3) are stitched into F(1-2-3) by using the optimal vertical offset *O_vo_*(2). Finally, after cycling the above steps, we stitch all of the images frame by frame together to complete the generation of a cylindrical borehole panorama. A diagram is of this is shown in Figure 3.

## 4. Experimental Results and Analysis

To verify the effectiveness of the proposed method, image stitching experiments were conducted on a natural scene image and a geological borehole image, respectively. The experimental equipment and conditions used in this paper were as follows: the CPU was the Intel Core (TM) i5-11300H, the highest main frequency was 3.10 GHz, the memory was 16 GB, the operating system was Windows 11 and the development environment was MATLAB R2020b. At this stage, the feature point extraction, image matching and stitching effect are analyzed to demonstrate the effectiveness of the proposed algorithm.

### 4.1. Analysis of Feature Point Extraction

In this section, we compare the feature point extraction results of the SURF algorithm using the natural scene image and the geological borehole image and perform the comparative analysis of the SIFT, Harris and ORB algorithms for the geological borehole image.

**Figure 4:**
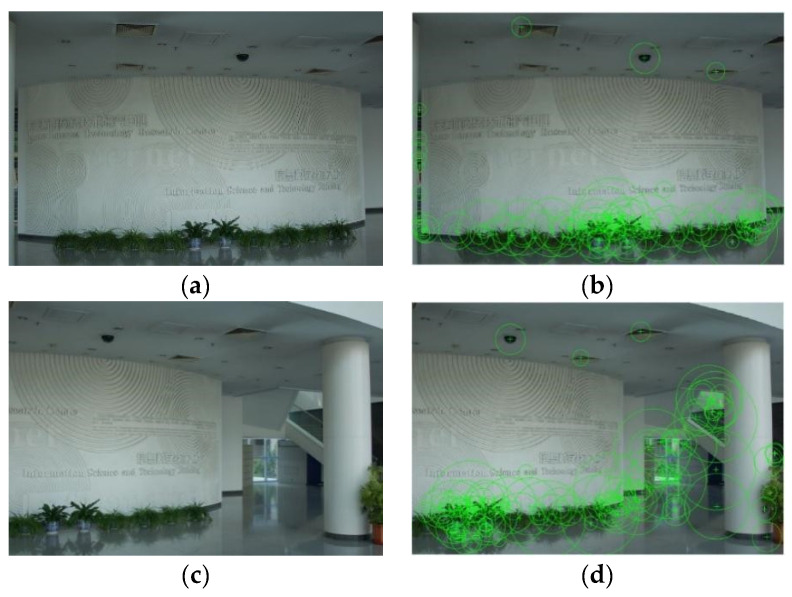
Feature points extraction results of natural images. (**a**) Nature image 1, (**b**) SURF feature points of natural image 1, (**c**) nature image 2, (**d**) and SURF feature points of natural image 2.

Figure 4a,c shows two scene images with overlapping areas taken using a hand-held camera under natural light, both of which contain 677 × 449 pixels. The SURF feature points of the two images are shown in Figure 4b,d, and correspondingly, 103 feature points and 139 feature points are extracted. It can be seen that these feature points are distributed more among the structure and texture corners of the image.

**Figure 5:**
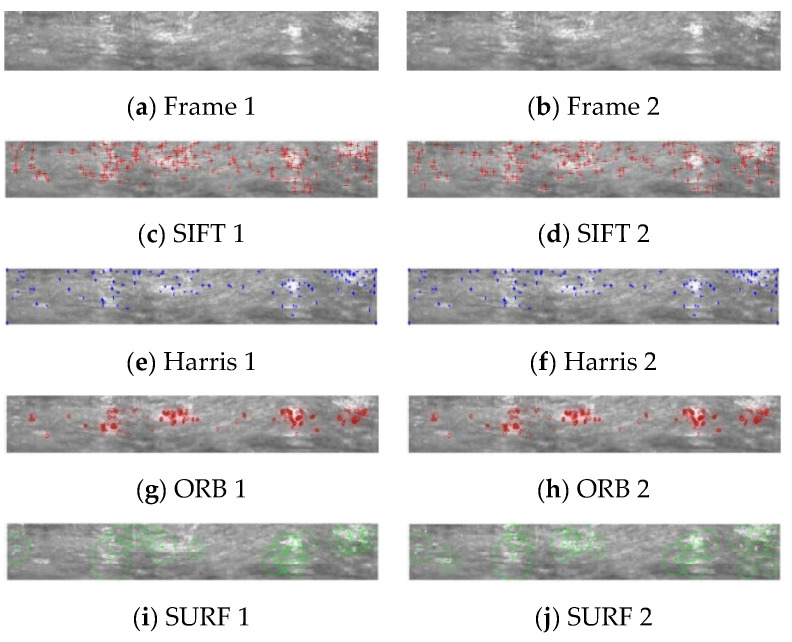
Feature points extraction results of 2 consecutive borehole unfolded images. (**a**) Borehole image 1 and (**b**) borehole image 2. (**c**,**e**,**g**,**i**) contain the SIFT, ORB Harris, SURF feature points of borehole image 1, and (**d**,**f**,**h**,**j**) contain the SIFT, ORB Harris, SURF feature points of borehole image 2.

Figure 5a,b presents the continuous two frame borehole unfolded images generated by the geological borehole video, which were taken using the APBCS, with the size of 512 × 64 pixels. The results obtained by extracting the feature points using the SIFT, Harris, ORB, and SURF algorithms are shown in Figure 5c through Figure 5j, respectively. The number of feature points extracted by the different algorithms and their running time for the two images are shown in Table 1. It is clear that the SIFT algorithm extracted the largest number of feature points, but the algorithm took longer to run. The number of feature points and the running time of the SURF algorithm selected in this paper can meet the stitching requirement of the borehole video.

It can be seen from the comparison with the natural scene image that the geological borehole image has a single color, minimal texture change and relatively few feature points are extracted. Based on the same algorithm, the number of feature points extracted from a single geological borehole image is reduced by about more than 100 compared with that achieved with the natural scene image, so it will be difficult to use this to effectively match the feature points and achieve high stitching accuracy. In general, the texture, color and local features of the natural images are much richer than those of the special geological borehole images. Therefore, the traditional algorithm meets the stitching demand between the natural images, but for the stitching of borehole images, it encountered problems of there being a few extracted feature points, a large matching rate error and a low stitching quality.

### 4.2. Analysis of Image Matching

In order to verify the matching accuracy of the proposed algorithm, under the condition that the parameters are consistent, the image matching accuracy analysis experiments are conducted on the natural scene image and the geological borehole images, respectively.

**Figure 6:**
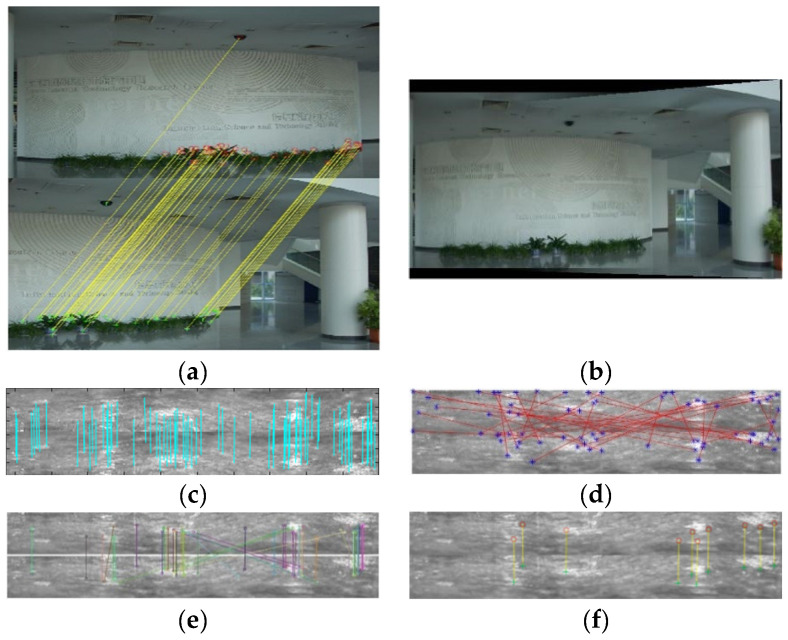
Matching effect of two images. (**a**) Nature image matching result; (**b**) nature image stitching result. (**c**–**f**) contain the borehole image matching results of SIFT, Harris, ORB and SURF algorithms, respectively.

Figure 6a shows the matching of feature points for Figure 4b,d after using the SURF and MASC algorithms. Figure 6b shows the stitching effect of the natural scene image by using the homography matrix, which was calculated using the matching pairs. The feature point matching of the SIFT, Harris and ORB algorithms and the algorithm in this paper for the two borehole images are shown in Figure 6 (c, d, e and f, respectively).

**Figure 7:**
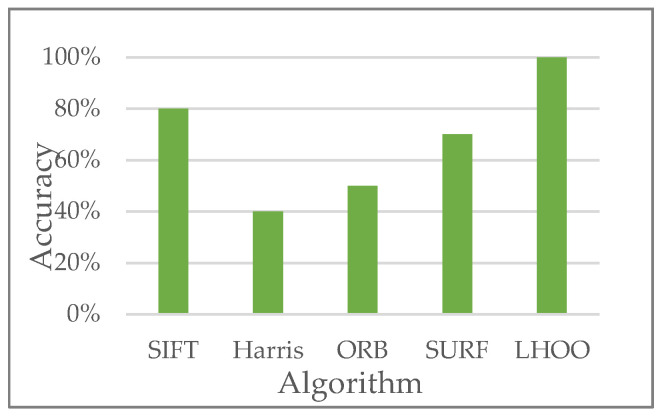
Matching accuracy of different algorithms.

In order to verify the performance of the SURF algorithm selected in this paper, based on the same experimental parameters, 10 matching tests were carried out on the same group of two consecutive rectangular unfolded images, which are shown in Table 2. In this test, the horizontal offset (*H_o_*) and vertical offset (*V_o_*) of the two images and the number of feature point pairs (*N_p_*) were counted, and the coordinate values of five feature point pairs on the two images are shown. It can be concluded that the data of the four matching tests are identical, the number of accurate matchings is seven and the mismatch rate is 30%. The optimal value of the vertical offset can be three, and the horizontal offset is approximately 0. For the above experiments, we also compare the 10 matching tests of the SIFT, ORB and Harris algorithms on the same group of unfolded images and obtain the matching accuracy (correct number/total number of tests), as shown in Figure 7. The results show that compared with the SIFT, Harris, ORB and SURF algorithms, the matching accuracy of our algorithm is improved by about 20%, 60%, 50% and 30%, respectively, thus ensuring the accuracy of the video stitching process.

**Figure 8:**
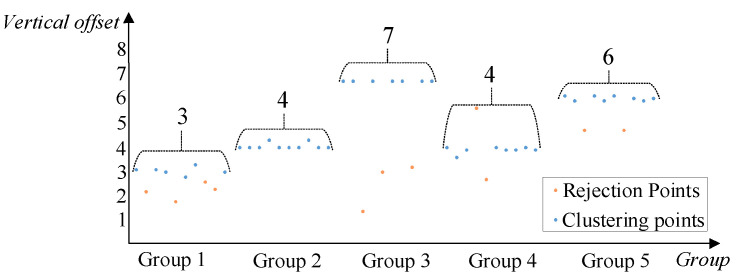
Statistics result of vertical offset.

To verify the accuracy of the LHOO algorithm for extracting the optimal vertical offset from different types of borehole unfolded images, five groups of unfolded images at different positions are selected for the ten vertical offset extraction tests. The statistical results are shown in Figure 8. It can be seen that the vertical offset will fluctuate up and down close to the optimal value at each time. The vertical offset value extracted from the second group belongs to the correct cluster point value, and the other four groups contain from two to four elimination points. After using the proposed LHOO algorithm proposed in this paper, the optimal vertical offset can be obtained accurately, which verifies the effectiveness of our algorithm.

### 4.3. Video Frame Stitching Effect

In order to verify the effectiveness of the video image stitching method proposed in this paper, in this stage, we conduct a real-time stitching experiment on 225 consecutive frames from the borehole unfolded images.

**Figure 9:**
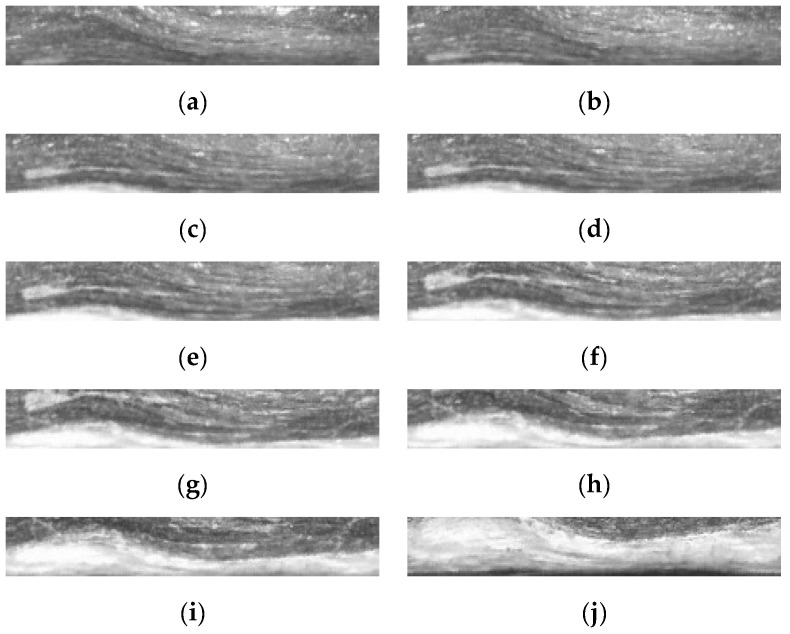
Selected 10 frame unfolded images from (**a**–**j**) for frames 1, 21, 43, 65, 85, 106, 127, 148, 169 and 210, respectively.

**Figure 10:**
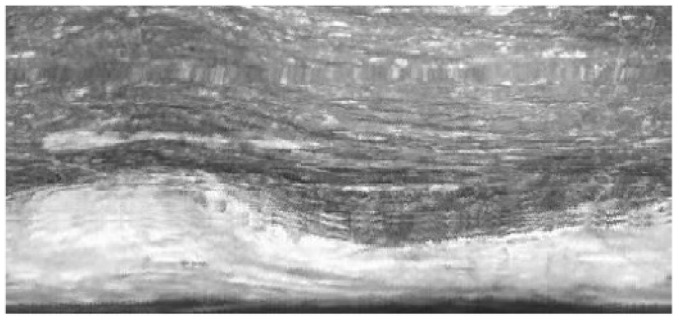
Panoramic view of drilling holes.

Figure 9 shows 10 samples of unfolded images at different frame bits selected from the borehole video sequence acquired by using the APBCS. We use the proposed LHOO algorithm to match and stitch 225 consecutive frames of borehole unfolded images, and finally, these images are fused into a complete borehole panorama, as shown in Figure 10. The average stitching time of each frame was 100 ms, which basically meets the requirements of the real-time system.

Our method is completely suitable for use on a geological borehole video that was obtained in a special environment, and it solves the problem of matching errors in low resolution images. The borehole panorama has a good visual effect, which also verifies that the stitching system can be applied to the actual geological drilling work environment.

The generation of the panoramic borehole image allows for us to perform a quantitative analysis of the direction, inclination angle, width and other borehole information of the structural surfaces, providing important reference values for intelligent image analysis in the geological drilling field, thus, improving the application potential of the forward-looking borehole camera system and providing important theoretical and application values.

## 5. Conclusions

In this paper, we propose an effective image sequence stitching method for a geological borehole video. After obtaining the homography matrix based on the SURF and MSAC algorithms, the LHOO algorithm is put forward to obtain the optimal vertical offset of two consecutive borehole images. Subsequently, our method is used for stitching the borehole image sequence to obtain a complete borehole panorama. The panorama obtained by the proposed method can clearly reflect the texture information of the borehole’s inner wall, and it meets the engineering visualization requirements. However, the method is a multiple offset statistical optimization type after obtaining the vertical offset, so it will increase the complexity of the entire algorithm, which will be inapplicable for some video real-time splicing systems which require a long running time.

For future works, the corresponding research will be conducted in the process of feature point extraction and matching, and the statistical optimization method used in this paper will be further improved to build a more rapid and accurate method to stitch the geological borehole video sequence. In addition, due to the image changes between the video frames, the stitching process will produce stitching seams. We will study how to effectively eliminate the stitching seams and further improve the stitching quality of the panorama.

## Figures and Tables

**Table 1:** Number of feature points and running time.

Algorithm	Nature Image			Borehole Unfolded Image		
	Left Image	Right Image	Time (ms)	Frame 1	Frame 2	Time (ms)
SIFT	468	385	1020	313	279	410
Harris	363	433	150	94	109	90
SURF	103	139	71	16	18	60
ORB	2541	2469	58	192	183	10

**Table 2:** Statistical results of match test.

*N*	*V_o_*	*H_o_*	*N_p_*	Feature Point 1		Feature Point 2		Feature Point 3		Feature Point 4		Feature Point 5	
1	3.1	0.4	10	(297,18)	(298,24)	(52,20)	(54,18)	(102,26)	(103,24)	(143,42)	(144,41)	(117,44)	(119,43)
2	2.2	2.2	9	(358,19)	(357,16)	(13,23)	(31,21)	(102,26)	(103,24)	(124,34)	(125,28)	(370,41)	(369,39)
3	3.1	0.3	7	(358,19)	(357,16)	(52,20)	(54,18)	(102,26)	(103,24)	(143,42)	(144,41)	(117,44)	(119,43)
4	3.0	0.6	10	(297,18)	(298,24)	(52,20)	(54,18)	(102,26)	(103,24)	(143,42)	(144,41)	(117,44)	(119,43)
5	1.8	2.6	8	(12,18)	(12,16)	(13,23)	(31,21)	(498,29)	(498,27)	(124,34)	(125,28)	(143,42)	(144,41)
6	2.8	0.2	9	(297,18)	(298,24)	(358,19)	(357,16)	(52,20)	(54,18)	(102,26)	(103,24)	(143,42)	(144,41)
7	3.3	0.4	10	(297,18)	(298,24)	(52,20)	(54,18)	(102,26)	(103,24)	(143,42)	(144,41)	(117,44)	(119,43)
8	2.6	0.3	9	(297,18)	(298,24)	(358,19)	(357,16)	(52,20)	(54,18)	(102,26)	(103,24)	(143,42)	(144,41)
9	2.3	1.8	7	(12,18)	(12,16)	(13,23)	(31,21)	(102,26)	(103,24)	(124,34)	(125,28)	(117,44)	(119,43)
10	3.0	0.6	10	(297,18)	(298,24)	(52,20)	(54,18)	(102,26)	(103,24)	(143,42)	(144,41)	(117,44)	(119,43)

## Data Availability

Not applicable.
